# Regional pedicled flap salvage options for large head and neck defects: The old, the new, and the forgotten

**DOI:** 10.1002/lio2.983

**Published:** 2022-11-29

**Authors:** Brent A. Chang, Ameya A. Asarkar, Peter M. Horwich, Cherie Ann O. Nathan, Richard E. Hayden

**Affiliations:** ^1^ Department of Otolaryngology – Head & Neck Surgery Mayo Clinic Arizona Phoenix Arizona USA; ^2^ Department of Otolaryngology/Head and Neck Surgery Louisiana State University Health Sciences Center & Feist‐Weiller Cancer Center Shreveport Louisiana USA; ^3^ Department of Surgery Overton Brooks Veterans Affairs Medical Center (OBVAMC) Shreveport Louisiana USA; ^4^ Head and Neck Specialists, HCA South Atlantic, Sarah Cannon Cancer Institute Charleston South Carolina USA

**Keywords:** pedicled flap, pedicled reconstruction, salvage reconstruction

## Abstract

**Objectives:**

The objective of this article is to review options for regional pedicled reconstruction for large head and neck defects in a salvage setting.

**Methods:**

Relevant regional pedicled flaps were identified and reviewed. Expert opinion and supporting literature were used to summarize and describe the available options.

**Results:**

Specific regional pedicled flap options are presented including the pectoralis major flap, deltopectoral flap, supraclavicular flap, submental flap, latissimus flap, and trapezius flap.

**Conclusions:**

Regional pedicled flaps are useful options in a salvage setting even for large defects and should be in the armamentarium of any reconstructive head and neck surgeon. Each flap option carries specific characteristics and considerations.

## Graphical abstract

This is a review article of different regional pedicled flaps for reconstruction of large salvage defects in the head and neck.

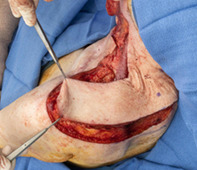

## 1 INTRODUCTION

In the era of modern head and neck reconstruction, microvascular free tissue transfer has become a widely utilized tool for many different types of defects. Before microvascular free flaps were pioneered decades ago, regional pedicled flaps were the primary option for reconstruction of large defects. The pectoralis major muscle flap, for example, became the workhorse flap used for many head and neck defects throughout the 1980s. By the 1990s the superiority of various free flaps for certain large defects had become apparent. This superiority of certain free flaps stems from various and different free flaps offering a closer match of donor tissue to missing tissue (bone, skin, muscle, and fat). However, free flaps still have their own inherent set of limitations. Unfortunately, a small number of free flaps fail. Risks of flap failure are well known, including poor vessel quality, significant medical comorbidity, infection, previous radiation, or other factors. While revision free flaps can always be entertained, the prudent surgeon must consider other options, especially if factors potentially related to the flap failure still persist. A famous quote usually attributed to Albert Einstein states, “The definition of insanity is doing the same thing over and over and expecting different results.” Vessel depleted necks, heavily radiated tissues, and severe atherosclerotic disease may all warrant consideration of a different reconstructive option. Regional pedicled flaps are an attractive option in the salvage setting even for large defects.

Certain pedicled flaps have unique advantages for certain defects, sometimes rivaling even the best free flap. The potential to provide reliable reconstruction, shorter surgery, and less resource‐intensive postoperative care cannot be ignored. Also, specialized operating room equipment, personnel, and postoperative care are usually not required for pedicled flaps. Color and texture match in the head and neck can be superior to free tissue transfer in certain circumstances. Advanced microvascular fellowship training is also not required for most regional pedicled flaps. Pedicled flaps do still carry some inherent potential drawbacks in general, as with any reconstructive option. Simultaneous flap harvest is not always feasible or practical. Contouring and placement of the flap can be more challenging in comparison to a free flap. Other controversial and often unspoken variables may also factor in to the willingness to perform pedicled flap reconstruction, from surgeon/facility compensation, to lost microvascular trainee benefit, and hopefully never the desire to have a “high free flap volume” (either as a department or individual) for perceived reputational value or resource allocation purposes.

In the setting of head and neck cancer, the term “salvage” can have a number of different meanings, particularly when talking about reconstruction. In this review, we are referring to salvage as reconstruction in the setting of any previous treatment. In reconstructive surgery, however, salvage typically refers to reconstruction after a previous reconstructive attempt has failed. Reconstruction in a secondary setting carries much higher risk.^1^

Despite the popularity of free flaps, numerous different regional pedicled flaps are still used in the modern era. While many different types of pedicled flaps have been described over many decades, some have fallen into disuse, while others have gained significant popularity in recent years. We describe and revisit in this review those common and uncommon regional pedicled flaps that have proven to be invaluable for salvage of large defects in the head and neck. These include: the pectoralis flap, the deltopectoral flap (“the old”), the supraclavicular flap, the submental flap (“the new”), the latissimus flap, and the trapezius flap (“the forgotten”). We describe modern modifications and descriptions for the use of these flaps in current head and neck reconstruction. While various other components of the reconstructive ladder exist to salvage head and neck defects (such as other free flaps, tissue expansion, local flaps, etc.), we focus here on regional pedicled flaps. In addition, while there are many other regional pedicled flaps that have proven to be valuable and need to be included in the armamentarium of the reconstructive surgeon, such as the facial artery musculo‐mucosal flap, sternocleidomastoid muscle flap, temporoparietal fascia/temporalis muscle flap, and palatal island flap, we will focus here on options that are particularly useful for large sized or large surface area defects.

## 2 THE PECTORALIS FLAP

Ariyan first described the pectoralis major pedicled musculocutaneous flap in 1979 and since then it has been a “workhorse” flap for head and neck reconstruction.^2^ Once used for all head and neck defects, the pectoralis major flap lost popularity when selected free flaps were shown to provide better donor tissue match in many defects. It remains, however, an excellent and versatile reconstructive option for some head and neck reconstruction.

**FIGURE 1:**
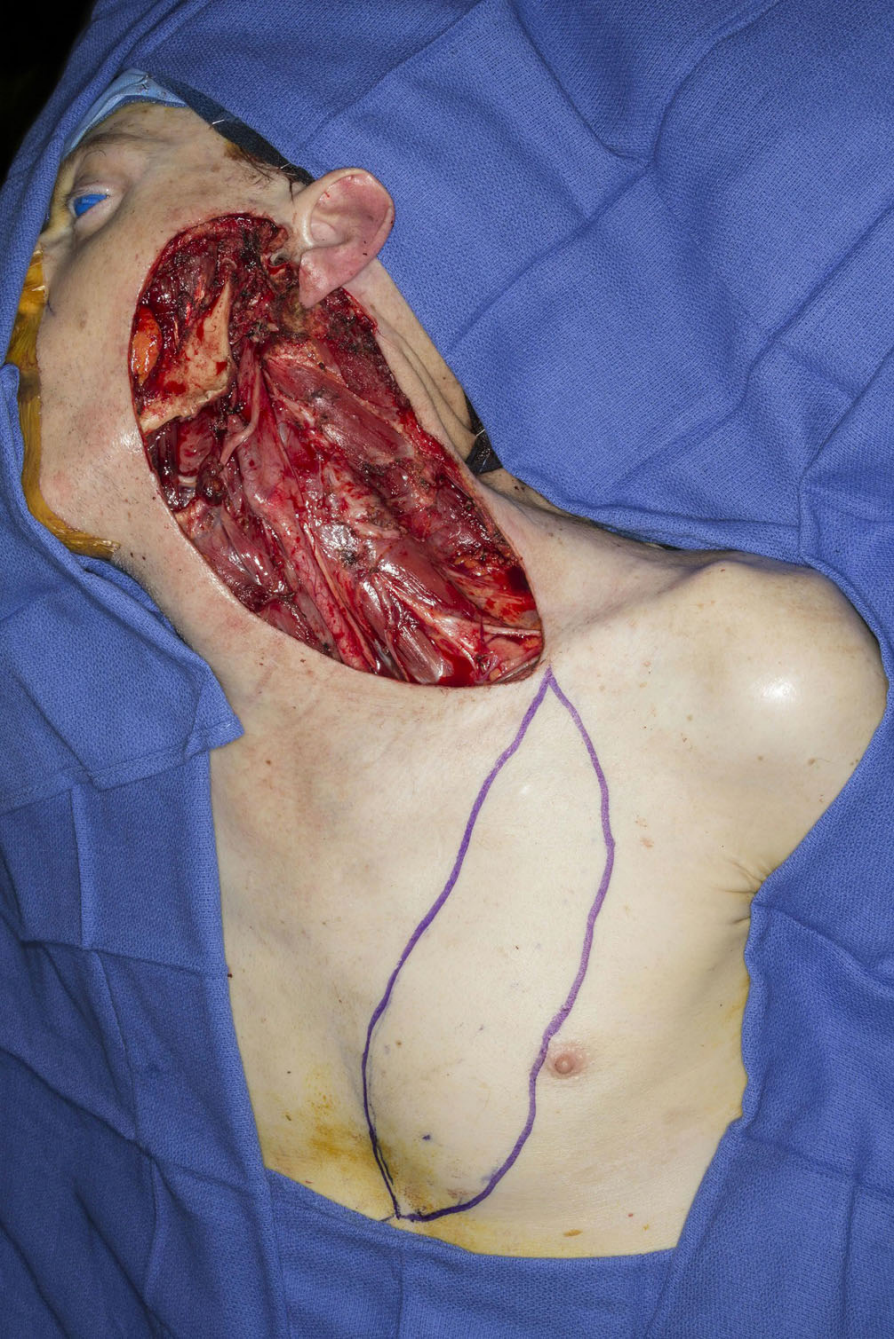
Pectoralis major flap (harvested)

The pectoralis major muscle is a large muscle originating from the medial third of the clavicle, sternum, the first six ribs, and the aponeurosis of the external oblique muscle (Figure 1). It inserts onto the greater tubercle of the humerus in the form of a single tendon. The main arterial supply for this flap is the pectoral branch of the thoracoacromial artery, a branch of the second portion of the axillary artery.

Large‐sized skin paddles can be reliably harvested over the pectoralis muscle. The pectoralis major flap can usually reach midface and lateral skull base defects, with a superior limit often being stated as the zygoma. The skin paddle can be reliably designed distal to the pectoralis muscle to improve reach by including undisturbed anterior rectus fascia with the random portion of the flap. While normally designed with a skin island directly over the inferior pectoralis muscle, a large elliptical otter tail design can be used to capture a higher number of perforators and maximize the possibility of providing blood supply to skin overlying the rectus fascia via the angiosome concept (Figure 1). Intraoperative fluorescence angiography imaging before and after flap elevation can help confirm the distal extent of viable random skin if necessary. A delay step could theoretically be used here to improve both distal reach and vascularity but is generally not recommended. Other modifications have been described to increase reach of the flap, such as removal of a portion of the clavicle and tunneling the flap underneath the clavicle.^3^, ^4^, ^5^ The skin paddle is designed to be located along the course of the pectoral branch of the thoracoacromial artery to maintain maximum blood supply through the perforators. The motor innervation to the flap is through the medial (C5–C7) and lateral (C8–T1) pectoral nerves. These nerves are typically transected to induce muscle atrophy and prevent muscle contraction.

Flap harvest is quick, easy, and reliable. The donor site can typically be closed primarily after undermining skin flaps over the anterior chest wall. The ability to primarily close the donor site can be quite variable depending on body habitus, donor site tissue quality, and willingness to undermine and distort nearby anatomy such as the nipple or breast. The flap is typically outside of head and neck radiation fields. The reliability of the pectoralis flap is one of its most attractive attributes. Various studies have showed less than 2% total flap failure and 7% to 9% partial flap failure.^6^, ^7^, ^8^ The harvest is straightforward and rapid, as meticulous pedicle dissection is not typically required.

**FIGURE 2:**
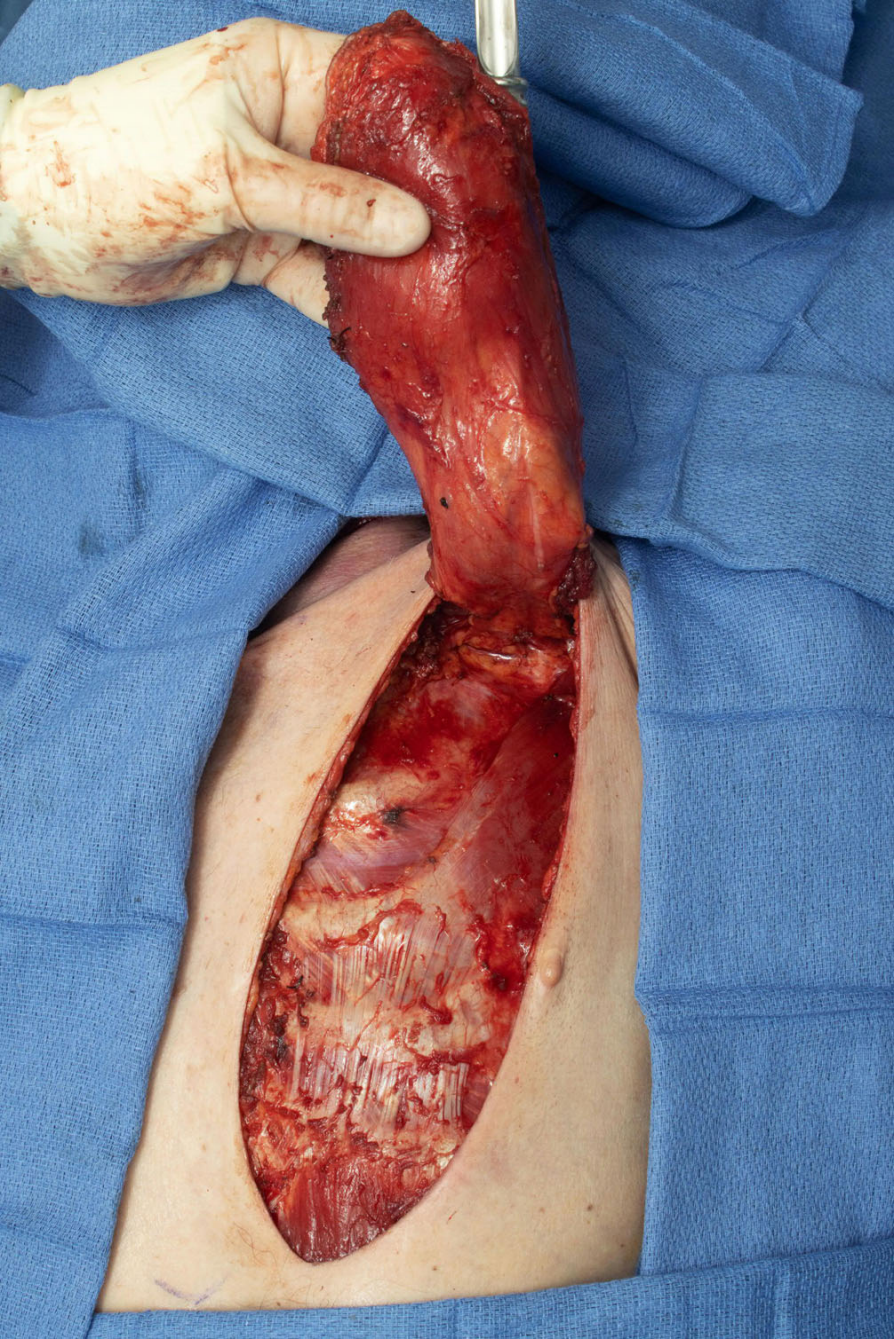
Pectoralis major flap (lifted)

**FIGURE 3:**
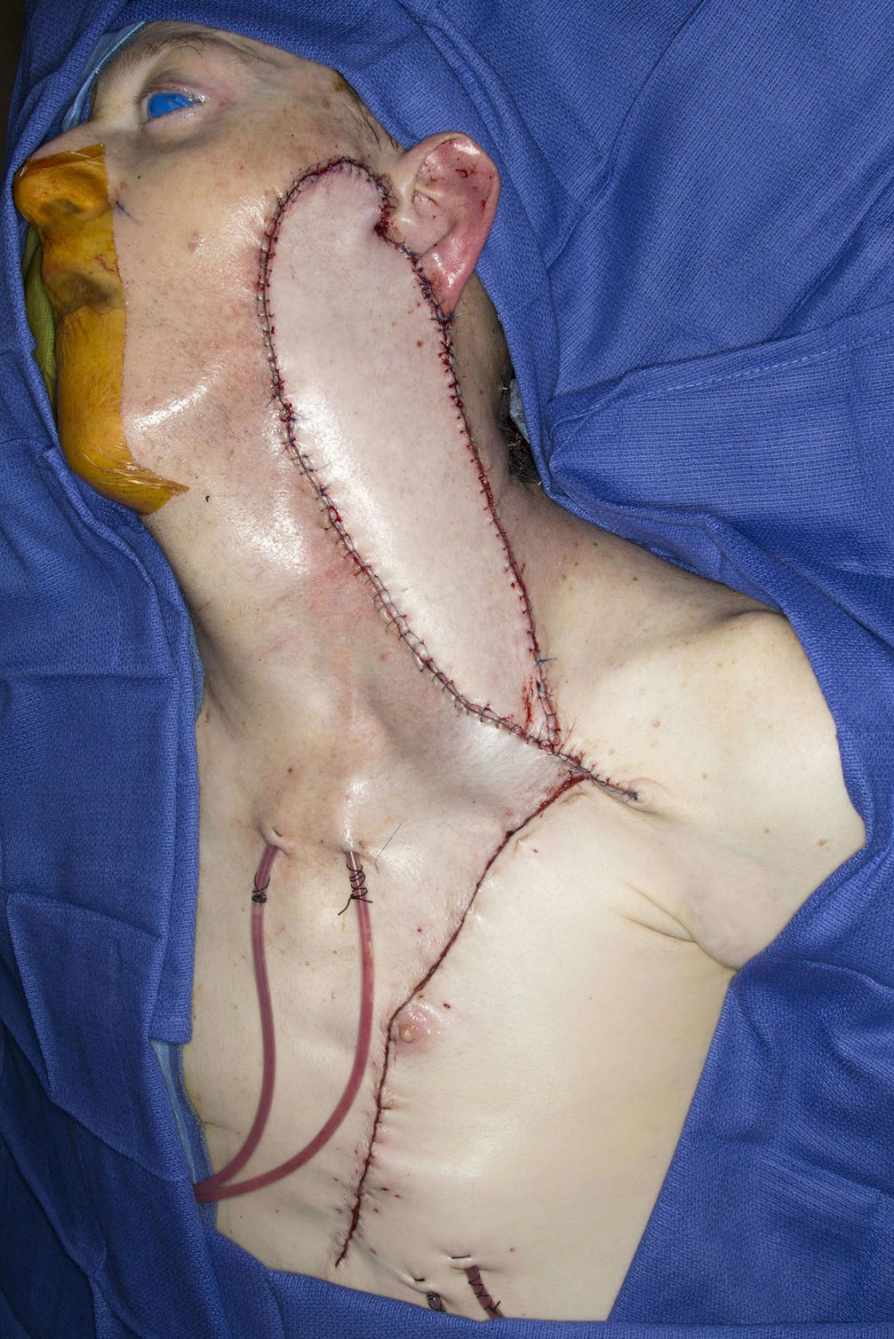
Pectoralis major flap (inset)

The pectoralis major flap possesses a number of characteristics, which can be advantageous or disadvantageous depending on the defect that is being reconstructed. The arc of rotation has a limit (Figure 2). The reach of the flap to higher head and neck defects can be challenging. The bulk is on the larger side, particularly in patients with large breasts or robust pectoralis muscles. Denervated pectoralis muscle will eventually atrophy, although fat and breast tissue will not. The large bulk makes the pectoralis flap excellent for situations in which there is a high risk of infection or leak, severely radiated tissues, or need for robust coverage such as in the setting of carotid exposure (Figure 3). The pectoralis major flap has limited versatility for tissue matching of different defects. Skin, fat, and muscle will all be harvested together unless a muscle‐only flap is used. As such, the pectoralis major flap has more utility in the setting of large volume defects and less utility for defects requiring thin lining for tissue match.

The donor site is also a consideration. There can be some cosmetic distortion from the bulk of the pedicle when tunneled through the neck and over the clavicle, although denervation of the flap does help with this. Distortion of the breast is common. Additionally, there is some associated shoulder dysfunction.^9^ Positioning and accessibility are usually favorable for most head and neck defects.

## 3 THE DELTOPECTORAL FLAP

The deltopectoral flap was first described by Aymard in 1917 for staged nasal reconstruction.^10^ Bakamjian popularized this flap in 1965 for pharyngoesophageal reconstruction following a total laryngopharyngectomy.^11^ However, it became a less popular option with the advent of pedicled musculocutaneous flaps such as the pectoralis major flap and microvascular free flaps.

Deltopectoral flaps are regional fasciocutaneous flaps based on the second, third, and fourth perforators of the internal mammary artery. The perforators demonstrate reliable blood supply from medial to lateral as far as the deltopectoral groove, beyond which the arterial supply is essentially a random pattern from the musculocutaneous perforators from the deltoid muscle. With a delay step removing this supply, the flap can reliably be harvested well beyond the deltopectoral groove. The venous drainage is through the venae comitantes of the internal mammary artery.

**FIGURE 4:**
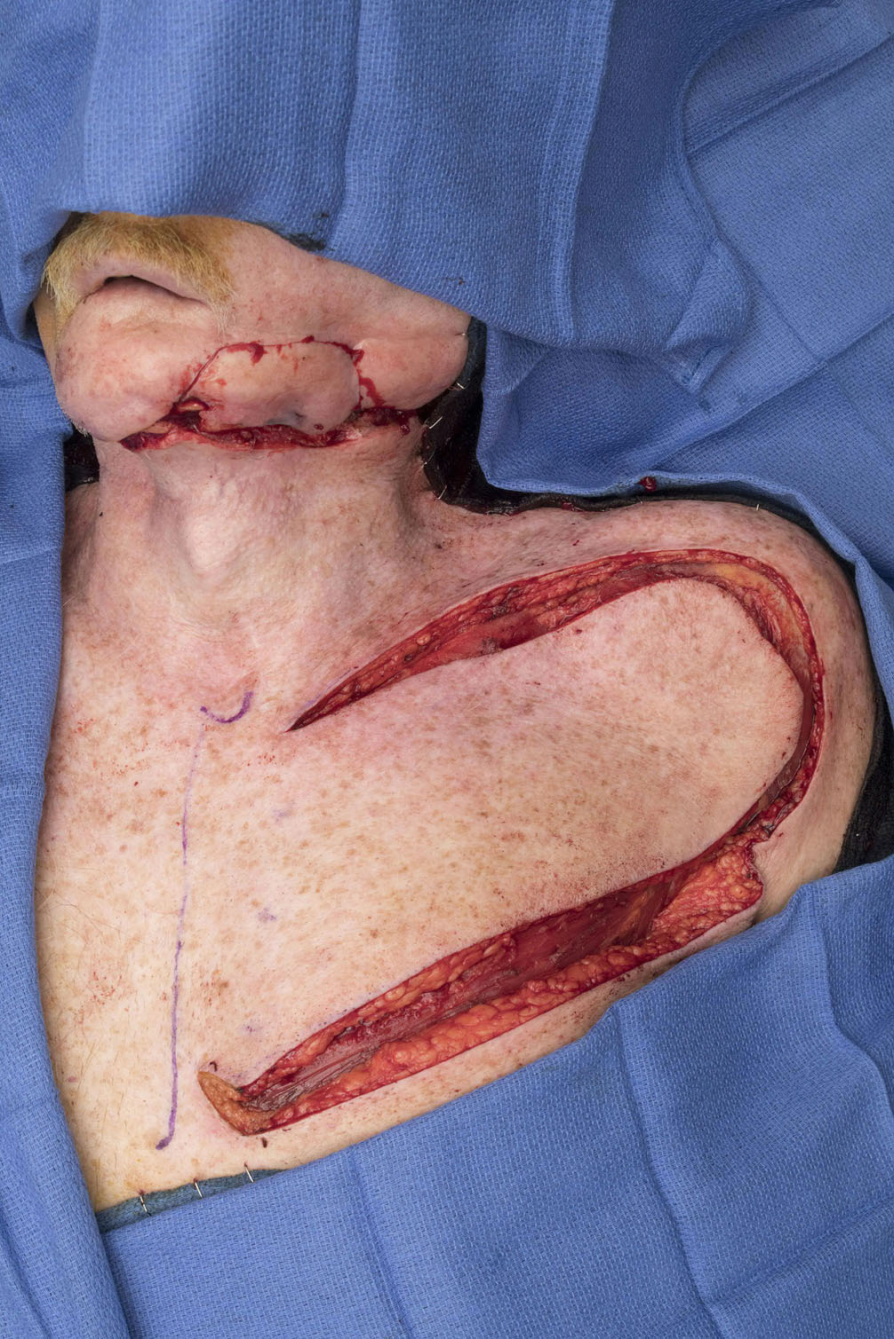
Deltopectoral flap (harvested)

The deltopectoral flap is extremely easy to raise and can be done in a rapid but safe manner. It provides a wide surface area with thin pliable tissue in most patients (Figure 4). The flap can be raised from 2 cm lateral to the sternum (to preserve the pedicle vessels) in the infra‐clavicular line to beyond the deltopectoral groove on the anterior shoulder. It is raised from distal to proximal, in a subfascial plane, including the fascia with the flap. The flap can be transposed directly with division of the intervening skin between the defect, in a tunneled fashion with de‐epithelialization of the proximal skin, or interpolated with division of the pedicle in a staged fashion.

If a delay step is not required for maximal pedicle length, caution is advised raising the flap beyond the deltopectoral groove due to the tentative blood supply.^12^ Inclusion of the investing fascia from the underlying pectoralis major helps protect the flap vascularity. The arc of rotation is leveraged on the inferior most internal mammary perforator.

**FIGURE 5:**
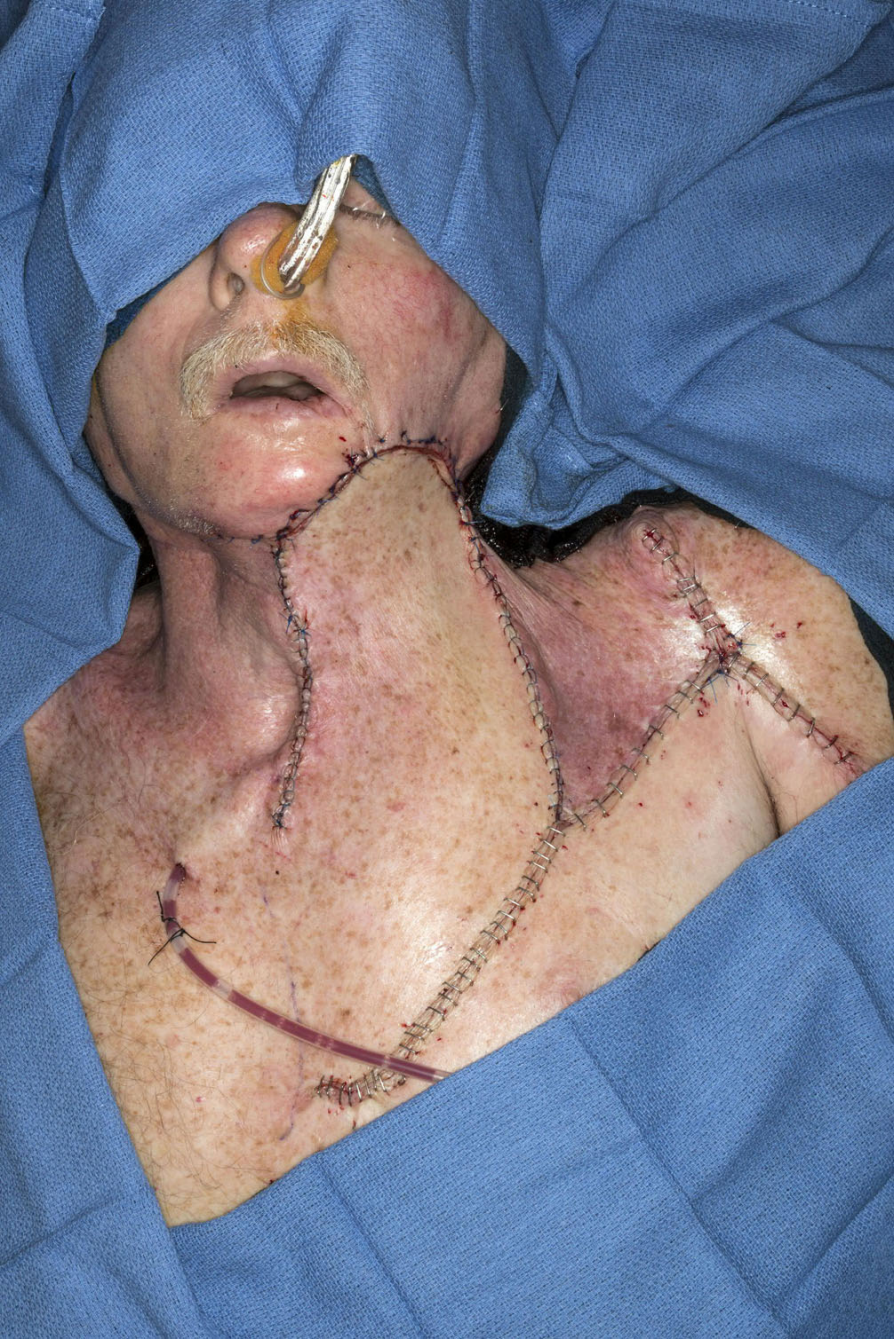
Deltopectoral flap (inset)

The deltopectoral flap has a quite limited arc of rotation for most head and neck defects. The reach of the flap is generally restricted to the neck or very low face, making this flap of limited utility for common head and neck defects such as oral cavity, oropharynx, and most facial defects. The deltopectoral flap typically has minimal bulk and thickness but can be harvested with a generous surface area (Figure 5). It has no option to incorporate muscle or other tissue types, limiting its versatility for tissue match purposes. It is an excellent option for resurfacing large surface area defects in the neck, particularly when large thickness reconstruction is not required.

The donor site can either be closed with wide undermining and primary closure or adjacent tissue transfer, or skin grafting which can add morbidity. In any case there is usually minimal functional deficit, as there is no muscle harvested in this flap. Positioning and accessibility are typically favorable for most head and neck defects.

There are a number of possible modifications. Bakamjian et al. described a medially based L‐shaped deltopectoral (L‐DP) flap which included extension of the flap over the anterolateral aspect of the arm.^13^ In this modification, back cuts are made medial and inferior to the 3rd internal mammary vessel perforator to increase arc of rotation. Chen et al. in 2012 compared the conventional deltopectoral to the L‐DP in head and neck reconstruction in 33 patients.^14^ The success rate and the complication rates in both groups were similar. L‐DP flaps are especially useful in full thickness head and neck defects which need to be folded to create an internal and external lining. We would advise a delay if such flaps are contemplated. The flap can also be modified into a cervico‐deltopectoral flap, in which a deltopectoral flap is mobilized as a large unit along with cervical skin and fascia (similar to a cervicofacial advancement flap). This is especially useful for resurfacing very wide defects extending up into the lower face or just beyond the reach of the traditional deltopectoral flap. This also avoids the need for tunneling of the flap. This arc of rotation can be improved slightly with the use of the internal mammary artery perforator flap (IMAP). This is a modification of the deltopectoral flap in which an islanded skin paddle supplied by a single IMAP is used.^15^ While the size of the potential flap is reduced with this modification, reports have suggested the IMAP flap can still be harvested up to 13 × 7 cm to 20 × 13 cm.^16^, ^17^, ^18^, ^19^

## 4 THE SUPRACLAVICULAR FLAP

The initial description of the precursor to the modern supraclavicular flap was in 1949 by Kazanjian and Converse as the “in Charretera” flap.^20^ Subsequent versions of this flap with skin paddles extending down to the elbow (the cervicohumeral flap) had poor distal reliability and never gained significant traction for head and neck reconstruction. The “supraclavicular artery island flap” was subsequently revived as a more proximally oriented island flap^21^, ^22^ and has since gained popularity for reconstruction of a variety of head and neck defects. The supraclavicular flap, like the deltopectoral flap, is a fasciocutaneous flap with thin, pliable tissue that is well suited for oral cavity and pharyngeal reconstruction but is also perhaps most useful for external cutaneous facial and neck defects due to the favorable color and texture match.

The flap is based off the supraclavicular artery, which is a branch arising from the transverse cervical artery. The venous drainage is through vena comitantes into the transverse cervical vein and the external jugular vein. The artery emerges from a triangle bordered by the posterior border of the sternocleidomastoid muscle, the external jugular vein, and the clavicle. The pedicle then usually passes transversely over the clavicle toward the deltoid muscle. The flap is designed centered over this course, with or without the aid of a Doppler ultrasound. The flap can generally be harvested up to 8 cm wide and still be closed primarily, although wider flaps are possible if the donor site is skin grafted.^23^ The relative ease of harvest and rapid nature in which the flap can be harvested make this an attractive option for many salvage defects.

**FIGURE 6:**
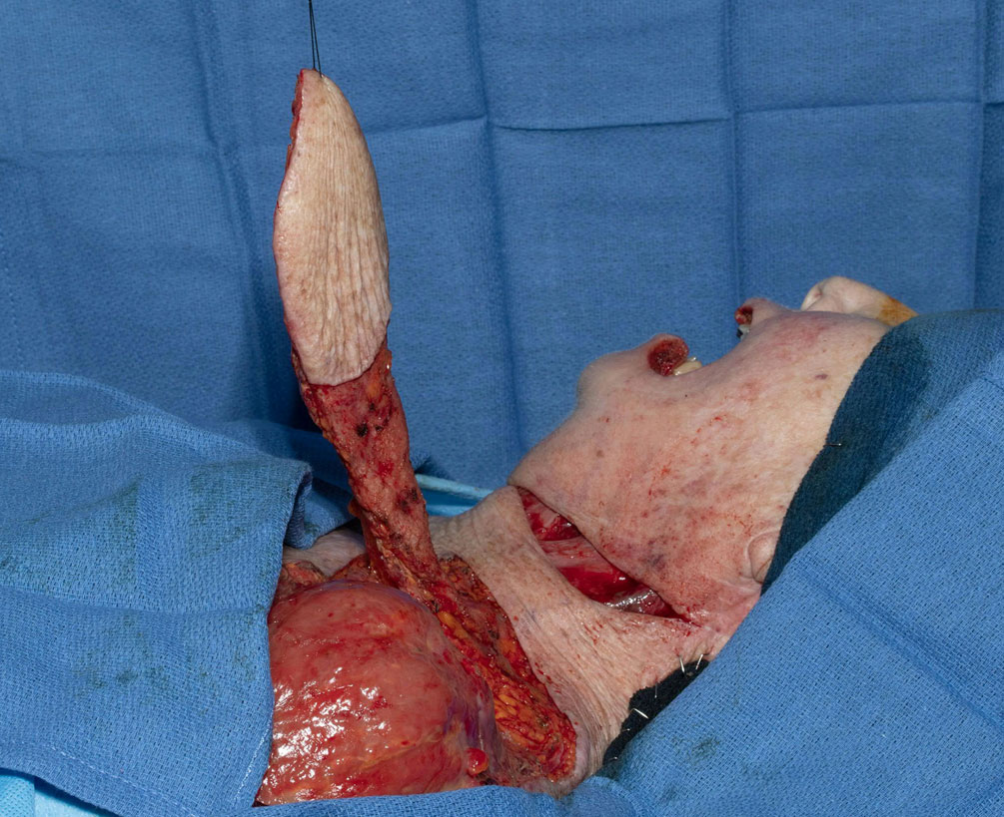
Supraclavicular flap (harvested)

**FIGURE 7:**
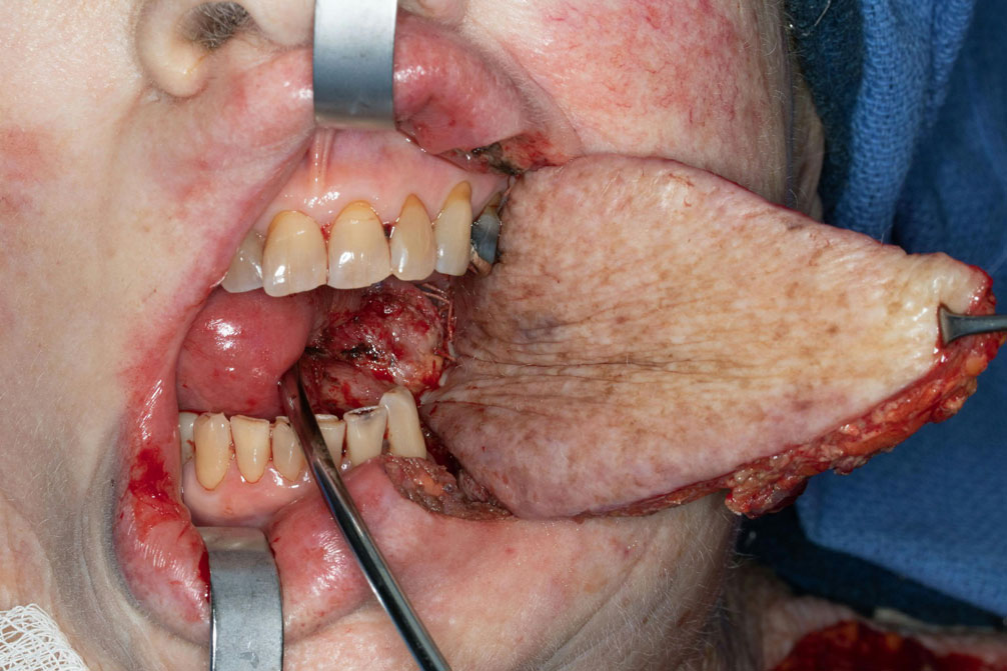
Supraclavicular flap (partially inset)

The arc of rotation is favorable for most head and neck defect locations (Figure 6). The flap will usually reach defects in the oral cavity, lower face, parotid, and lateral skull base with relative ease. Depending on anatomic and other factors, slightly higher defects can be reached with technical modifications such as delaying a longer flap but may have an increased risk of distal flap necrosis. Most patients have limited skin and subcutaneous thickness over the supraclavicular area which dictates the flap bulk. That thickness is often comparable to a thick radial forearm free flap, making it useful for oral cavity salvage (Figure 7). This flap is more useful for lining or coverage than for volume replacement. With excess length, the flap can be de‐epithelialized and folded on itself for modest improvement in thickness. Limited width can be problematic for especially large surface area defects if one wants to avoid skin grafting of the donor site.

Tissue match for more complex defects is somewhat limited. Generally, only fat and skin can be carried with this flap. While vascularized clavicle can in theory be transferred, the bone does not have enough reach to be useful for almost all defects in the head and neck. The lack of muscle also makes it less ideal for sealing oral or pharyngeal defects following chemoradiation. The color and texture match of the supraclavicular flap for facial defects is generally superior to distant free donor sites and regional pedicled donor sites on the torso.

Functional deficit from this donor site is also minimal, as there is no disruption of the shoulder musculature. Tightness in the immediate postoperative period from wide flaps usually resolves fairly quickly.

Studies have shown the supraclavicular flap to have good outcomes for lower facial, parotidectomy, auriculectomy, and lateral skull base defects.^24^ Alves et al. specifically studied the use of the supraclavicular flap in a salvage setting, demonstrating good reliability in this context.^25^ In a salvage setting, great care needs to be taken to ensure that there has been no disruption of the transverse cervical vessels in the previous surgery. A previous level IV neck dissection is not a direct contraindication to supraclavicular flap harvest; but a previous level V neck dissection assures less confidence. Computed tomography angiography may be useful if the status of the transverse cervical vessels cannot be reliably obtained from other imaging or operative reports. Previous shoulder surgery may also limit the use of this flap.

Proper design of the skin paddle is an essential element to success. While not absolutely critical, Doppler marking of the flap pedicle is a useful adjunct that will improve the reliability and comfort levels in surgeons new to this flap. The flap can be harvested as a distal skin island with proximal epidermal elevation.^26^ However, it is sometimes easier to simply design a long skin paddle encompassing the proximal pedicle origin, and then de‐epithelialize the proximal skin as needed.

A modification of the supraclavicular flap is possible based on an anterior branch of the supraclavicular artery which crosses over the clavicle medially. This flap has been given multiple different names in the literature, including: infraclavicular flap, cervico‐pectoral flap, pectorally extended supraclavicular flap, anterior supraclavicular artery perforator (a‐SAP) flap, and helicopter flap.^27^, ^28^, ^29^, ^30^ While the nomenclature is heterogeneous, these all refer to the same concept. The flap itself is similar in tissue quality to a traditional supraclavicular flap and carries similar qualities and considerations to those already discussed.

## 5 THE SUBMENTAL FLAP

First described in 1993 by Martin, the submental flap (or submental island flap, submental artery island flap) is a versatile, robust locoregional flap which for certain defects may be equal or superior to free flaps.^31^ This flap can be harvested as a cutaneous, musculofascial, or musculocutaneous flap and is based on the submental branch of the facial artery and the submental vein of the facial venous system. The submental flap has a wide arc of rotation that can reach defects throughout the oral cavity, oropharynx, parotid bed, and face. Coverage of lateral temporal bone and orbital exenteration defects has also been described.^31^, ^32^, ^33^, ^34^ Available skin paddle size depends on patient body habitus and can vary widely – submental flaps as large as 17 cm × 7 cm have been described in the literature.^31^, ^35^

**FIGURE 8:**
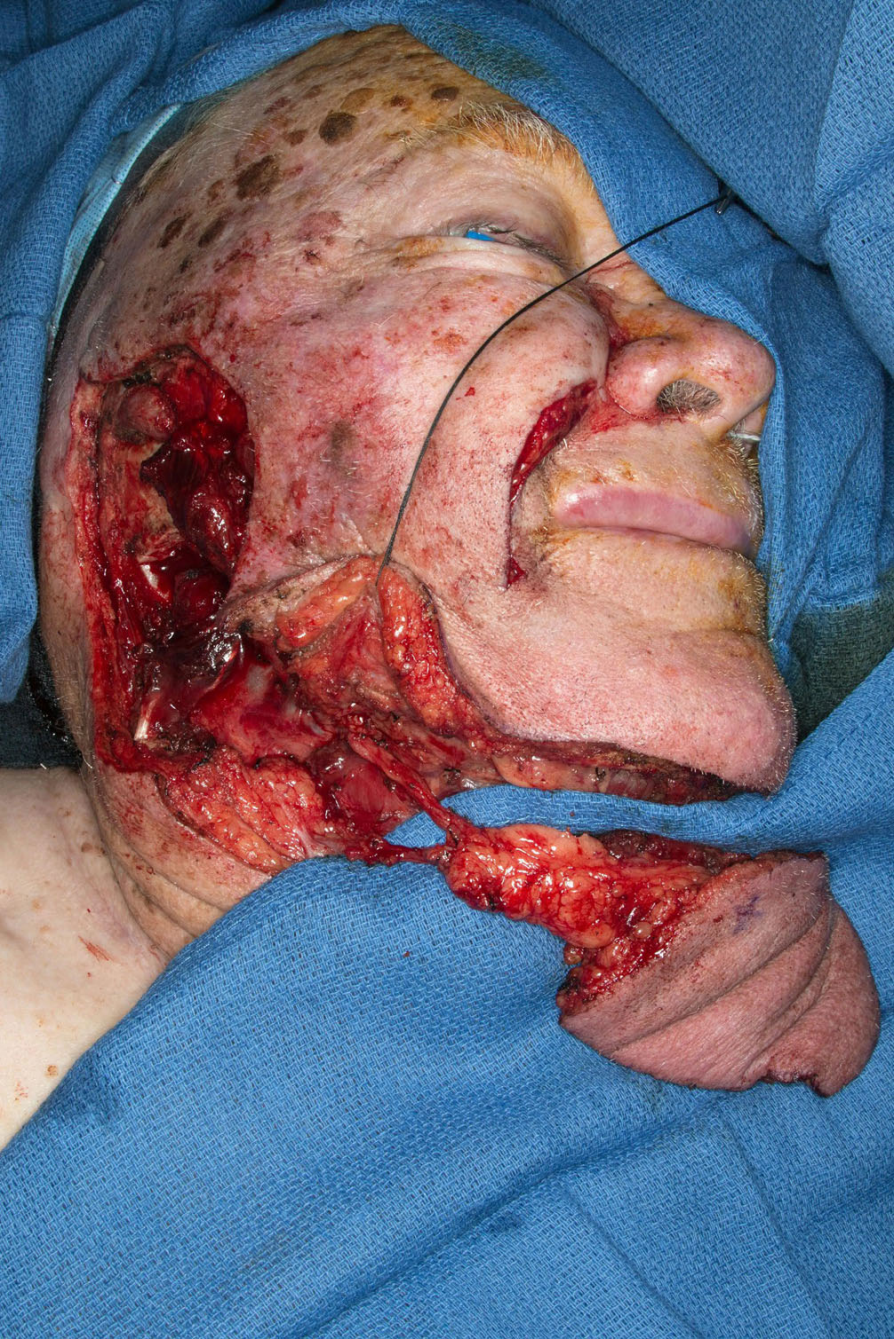
Submental flap (harvested)

**FIGURE 9:**
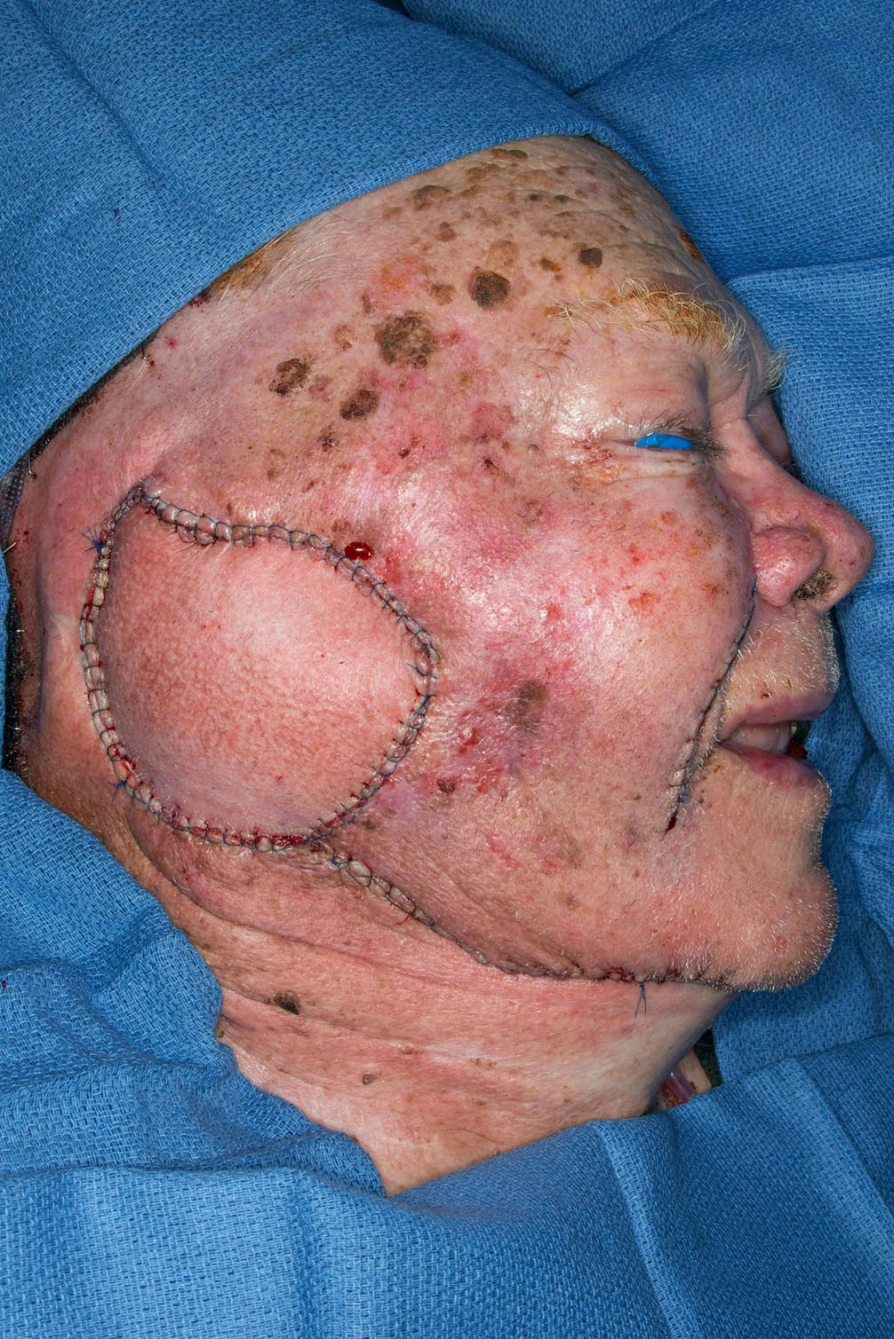
Submental flap (inset)

The submental flap has an excellent arc of rotation for most head and neck defects. The reach is sometimes limited for higher facial defects, although it can reach orbital, high facial, and anterior skull base defects in patients with favorable anatomy.^36^ The flap can also be “hybridized” by dividing and re‐anastomosing the pedicle vein to improve the reach.^37^ The potential to harvest reverse flow flaps can improve reach in certain circumstances. The submental flap is typically a thin and pliable flap with variable amount of submental fat but can also be customized for volume matching of different defects (Figure 8). It can be harvested with additional bulk by taking one or both anterior belly(s) of the digastric muscle(s) and the entire mylohyoid muscle. The submental flap excels in its versatility for tissue matching purposes. Different tissue components (skin, fat, muscle, bone) can be harvested in almost any combination. Although not well described in the literature, consistent anterior nutrient arterial branches of the submental system provide vascular supply to the bony mentum of the mandible, which can be incorporated into the submental flap to provide a short bony segment that is useful for reconstruction of the premaxilla and orbital rim. The submental flap can also be useful for repairing pharyngeal defects. It can be used as a musculocutaneous flap in pharyngectomy or laryngectomy defects, in which the muscle component is helpful for addressing the potential for pharyngeal leaks. The flap can also be used without skin as a musculofascial flap for reinforcement of primarily closed pharyngeal repairs.^38^ It has an unparalleled facial skin color and texture match (Figure 9). It can be harvested as a thin pliable piece of tissue which can be favorable for many oral cavity and facial defects.

The minimal donor site morbidity and accessible location of the donor site within the head and neck are also favorable features. A large flap skin paddle can be incorporated into a standard neck dissection incision if required, allowing for primary closure with minimal donor site morbidity, making the submental flap an excellent choice during salvage cases so long as the submental vascular pedicle has not been compromised by previous surgery. When use of this pedicled flap provides equal outcomes to a free flap, using the submental flap has been shown to decrease hospital length of stay and also offers a cost reduction.^33^, ^35^

The submental flap demands an oncologically sound level I neck dissection during harvest if being employed for oral cavity reconstruction. This oncologically sound approach has been extensively studied in oral cavity malignancy reconstruction demonstrating no difference in locoregional control or recurrence rates.^35^, ^39^, ^40^, ^41^, ^42^, ^43^, ^44^, ^45^ Caution is always advised in cases with level I lymphadenopathy, particularly if there is a concern for extracapsular extension.

A novel harvest technique was published in 2007 by Patel et al.^46^ and expanded on by Zenga et al. in 2019,^47^ emphasizing the incorporation of the ipsilateral mylohyoid muscle and anterior belly of the digastric in the flap pedicle. This simple, yet effective modification further protects the distal submental artery and dominant cutaneous perforators leading to greater flap viability and decreased flap tip necrosis.

## 6 THE LATISSIMUS DORSI FLAP

The first description of the latissimus flap for pedicled head and neck reconstruction was by Quillen et al. in 1978.^48^ The latissimus dorsi flap is currently more commonly used as a free flap in head and neck reconstruction; however, it can also be used as a pedicled flap. This flap is based off the thoracodorsal artery and vein, which are components of the subscapular vascular system. The thoracodorsal artery extends from the axilla inferomedially in a plane deep to the latissimus dorsi muscle. It is typically used as a musculocutaneous flap but can also be used as a musculofascial flap. The latissimus dorsi is a wide, flat muscle with the largest surface area of any muscle. It inserts into the humerus. It originates from the thoracolumbar spine, iliac crest, and ribs.

**FIGURE 10:**
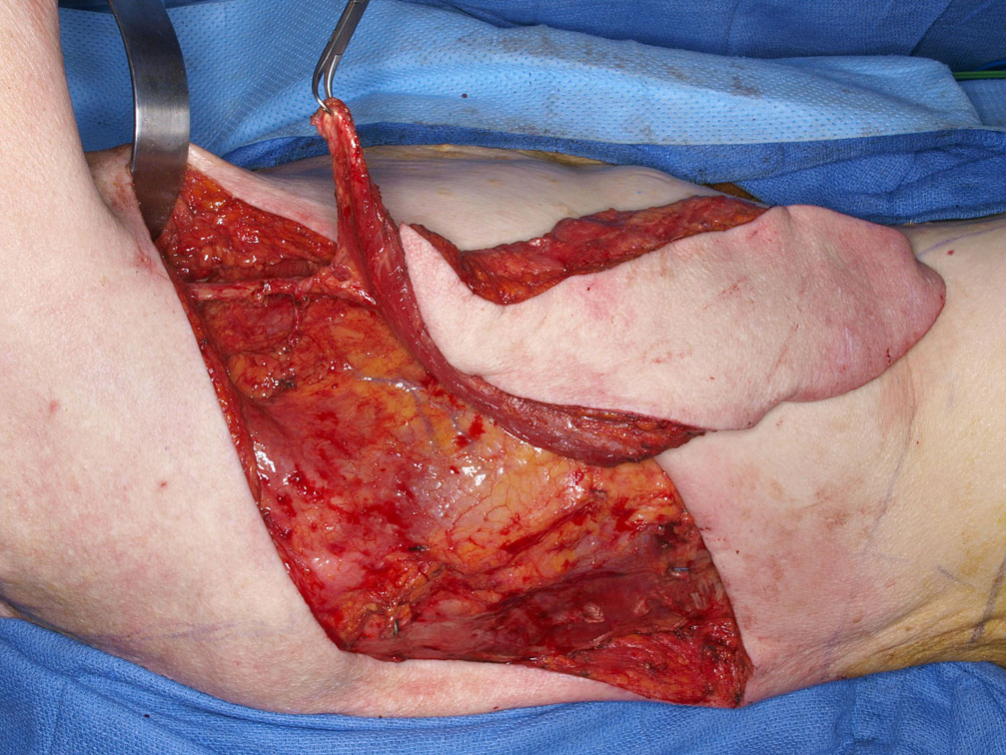
Latissimus dorsi flap (harvested, towel clamp on cut humeral tendon)

The pedicled latissimus dorsi flap is an especially useful option for salvage reconstruction of large head and neck defects. When used as a pedicled flap, the latissimus still has excellent reach; this is often an area of concern when using regional pedicled flaps for large head and neck defects. The extended length allows it to reach defects high on the head and the entire neck. The abundant available muscle and potential for a wide skin paddle make it good for resurfacing large surface area cutaneous defects and for filling defects requiring a large amount of bulk (Figure 10). The bulk of the entire latissimus dorsi flap can sometimes be challenging to tunnel, fit, and contour to certain defects in the head and neck. It can provide a very large surface area skin paddle that can still usually allow primary closure at the donor site. The surface area is unrivaled among pedicled flaps. There is some versatility in the type of tissue taken with this flap. It is possible to harvest this as a muscle‐sparing or even thoracodorsal artery perforator (TDAP) flap, although this is uncommonly done for head and neck salvage reconstruction as the arc of rotation of this variant is limited. There is also flexibility in the amount of skin included and it can also be used as a purely musculofascial flap. Vascularized rib can also be included but has limited practical utility.

The donor site morbidity from latissimus dorsi muscle harvest is generally favorable.^49^ Positioning to harvest a latissimus flap is an important consideration. Usually, lateral decubitus or semi‐lateral decubitus positioning are needed, which often prolongs operative time. The latissimus flap can be harvested from a supine position; however, this can be tricky and is not always feasible, particularly in patients with large body habitus.

**FIGURE 11:**
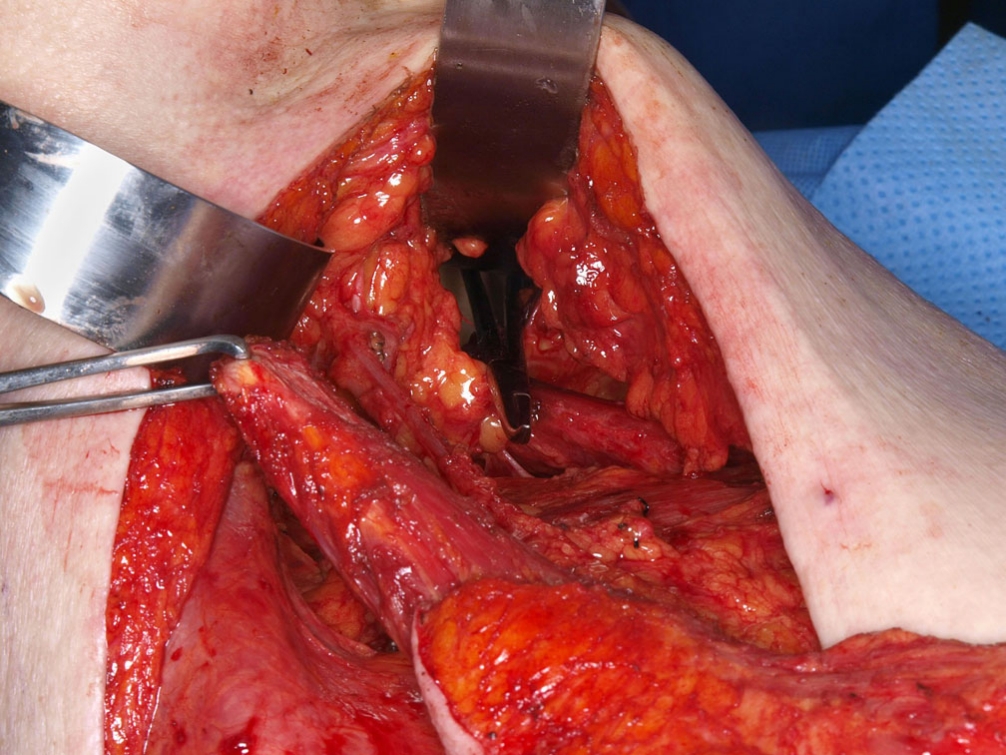
Latissimus dorsi flap (close up of axilla). Figure shows hemostat placed within tunnel, Allis clamp on humeral tendon with flap is still on the back prior to passage of flap through tunnel

**FIGURE 12:**
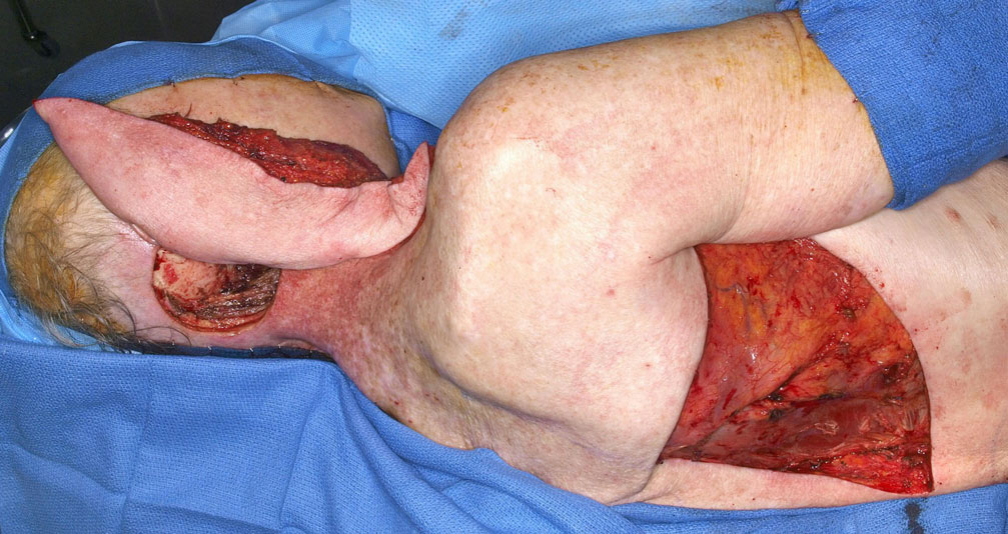
Latissimus dorsi flap (harvested). Flap has been passed through axillary tunnel

The use of the pedicled latissimus dorsi flap for most head and neck reconstruction involves tunneling the flap through the axilla. The vascular pedicle is dissected to the axillary vessels. The latissimus is then tunneled from the axilla to the neck (Figure 11). The tunnel begins above pectoralis minor and below pectoralis major if it is still present. A portion of the clavicular head of the pectoralis major is sectioned lateral to the thoracoacromial vessels to allow a tunnel 4 fingerbreadths wide. Tunneling of the flap through the axilla still allows good reach to the posterior neck and scalp, as demonstrated in Figure 12. While the flap can in theory be swung posteriorly without tunneling through the axilla, this actually reduces superior reach of the flap. The latissimus dorsi can still be used if a previous pectoralis flap has been harvested, which makes the tunneling stage easier. Conversely, a pectoralis flap can still be utilized after a previous pedicled latissimus dorsi flap but only if the thoracoarcomial pedicle has been preserved. The donor site has rarely been violated in most head and neck patients. As such, this flap more than any other is especially useful when more common salvage reconstructive options such as the pectoralis major flap have already been performed—it is a great “salvage salvage” flap.

**FIGURE 13:**
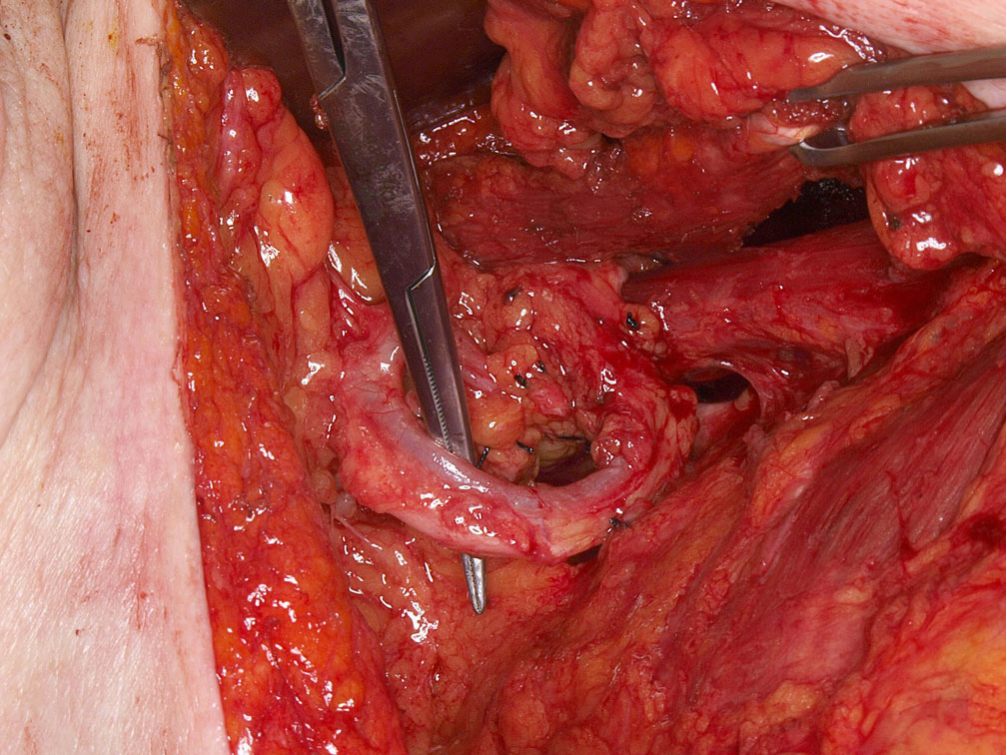
Latissimus dorsi flap (close up of axilla). Figure demonstrates vascular pedicle with gentle arc of rotation, flap passed through axillary tunnel until desired pedicle geometry achieved, Allis clamp on humeral tendon

**FIGURE 14:**
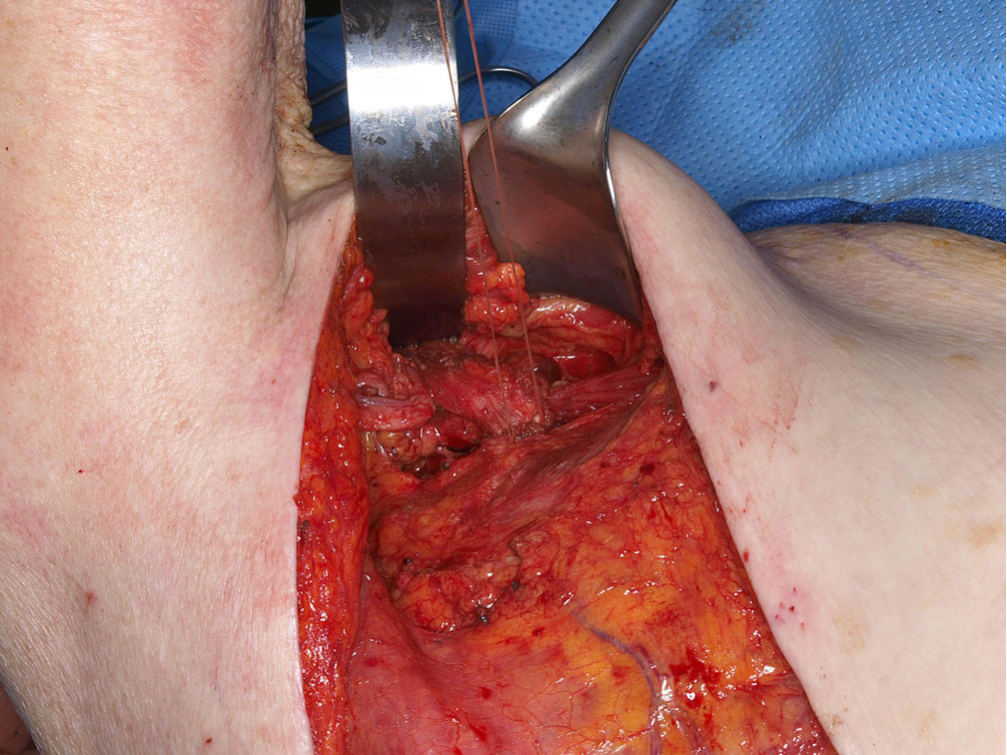
Latissimus dorsi flap (close up of axilla). Figure demonstrates two sutures anchoring humeral tendon to rib periosteum

**FIGURE 15:**
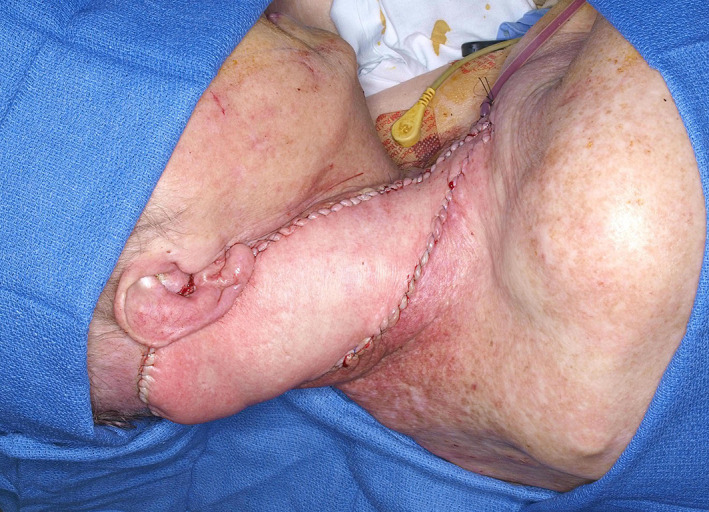
Latissimus dorsi flap (inset)

When used as a pedicled flap, there are several modifications that have been shown to improve reliability.^50^ Briefly, these modifications include (1) an otter tail skin paddle design (Figure 10), (2) preservation of the circumflex scapular vessels, and (3) fixation of the muscle's humeral tendon to rib periosteum. The fusiform otter tail design captures cutaneous perforators from the proximal angiosomes of the latissimus dorsi. This ensures superior vascularity to the distal skin flap. Preservation of the circumflex scapular vessels (Figure 13) and fixation of the tendon to rib periosteum both help avoid kinking of the proximal vascular pedicle (especially the vein) (Figure 14). These key modifications allow for reliable vascularity and flap survival (Figure 15). The skin paddle can be easily de‐epithelialized as necessary.

## 7 THE TRAPEZIUS FLAP

The trapezius is a large triangular shaped muscle extending from the occiput to the thoracic vertebrae, providing a large anatomic area with various different blood supplies that have been utilized for flap designs. Three different pedicled trapezius flaps are available, each based on a different vascular supply. A number of different authors have contributed to the original descriptions of the trapezius flap and its variations.^51^, ^52^, ^53^ In the literature, even the nomenclature for this flap system is confusing and variable. It is perhaps not a surprise that this is a “forgotten flap” not commonly used by most head and neck reconstructive surgeons in the modern era. Simplified these can be described as: the superior trapezius flap, the lateral island trapezius flap, and the lower island trapezius flap. The trapezius flaps have different blood supplies, depending on the specific type of flap. The superior trapezius flap is supplied by the paraspinous perforators and also receives contribution from the occipital artery. The lateral island trapezius flap and the lower island trapezius flap are supplied by the transverse cervical vessels. The lower island trapezius also receives contribution from the dorsal scapular artery if based inferiorly enough. The blood supply of the lateral and lower island flaps can be highly variable, with different variations arising from the thyrocervical trunk and subclavian artery, along with different relations to the brachial plexus. A detailed discussion of this is beyond the scope of this article.

**FIGURE 16:**
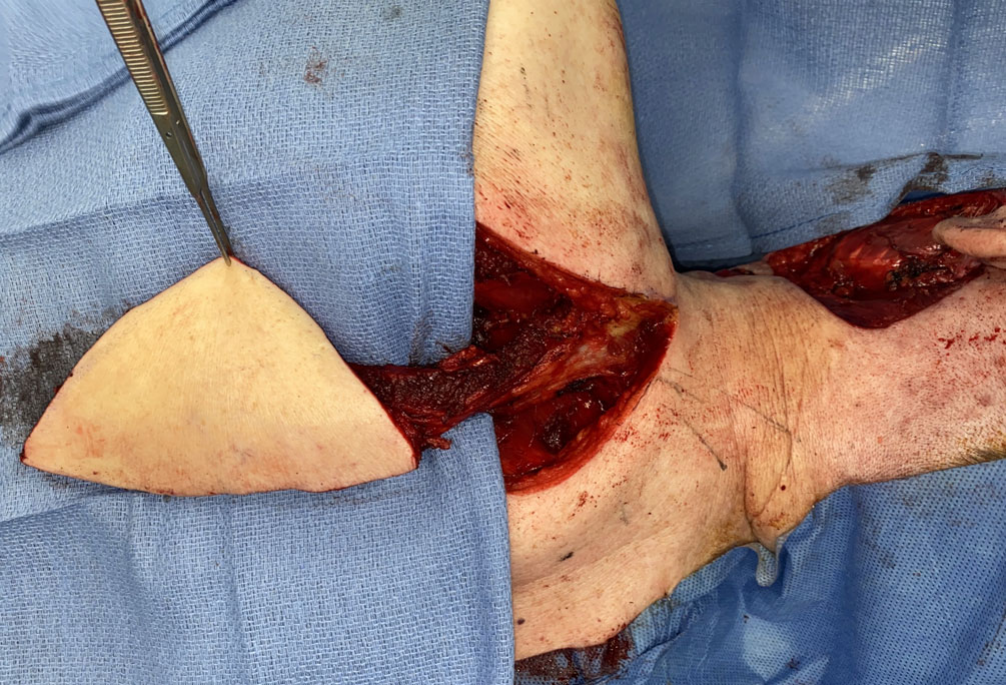
Lower island trapezius flap (harvested)

**FIGURE 17:**
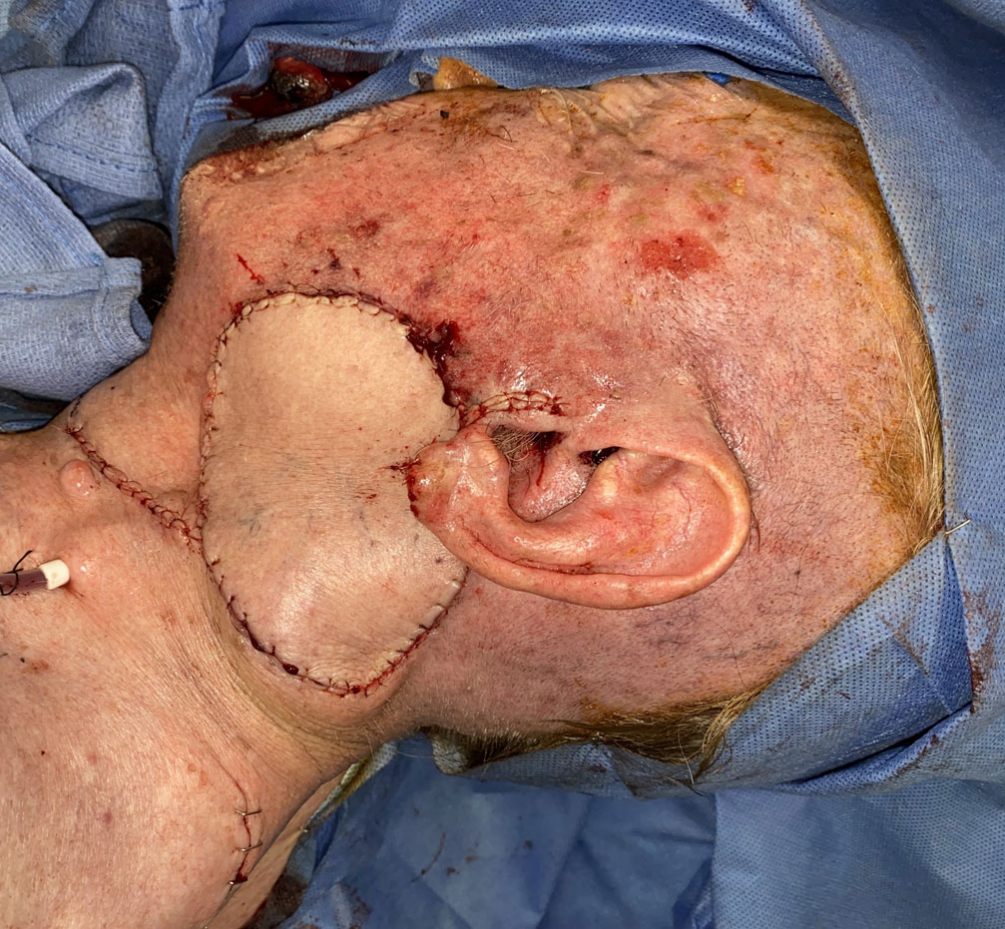
Lower island trapezius flap (inset)

The lower island trapezius flap has respectable reach to the head and neck, as it can be harvested even inferior to the trapezius muscle in the back (Figures 16 and 17). However, inferior extension of the skin paddle below the trapezius muscle (which can be assessed before and after flap elevation with intraoperative fluorescence angiography to confirm good skin vascularity) generally requires preservation of the dorsal scapular artery and vein. The lateral island trapezius flap can also reach most defects in the neck, lower lateral face, and lateral skull base (Figure 6). The superior trapezius has limited reach but is also superiorly based, making if favorably located for some defects in the head and neck, particularly lateral and posterior defects. It has the added benefit of not being gravity dependent, which is not the case for most regional pedicled flaps used in head and neck reconstruction. All three flaps have a moderate amount of bulk and can be modified to a degree by including more or less muscle. Versatility for tissue matching is somewhat variable. Each of the three generally needs to carry muscle, fat, and skin, although musculofascial flaps are an option. Bone from the scapular spine can be carried with the lateral island trapezius flap.

Positioning can be seen as a potential limitation for trapezius system flaps. Semi‐lateral decubitus positioning or other innovative methods can be used to maximize operative efficiency, but full lateral decubitus or even prone positioning may be required for harvest of lower island trapezius flaps. Shoulder dysfunction, as a result of harvesting trapezius muscle, is another potential drawback of all the trapezius system flaps.

**FIGURE 18:**
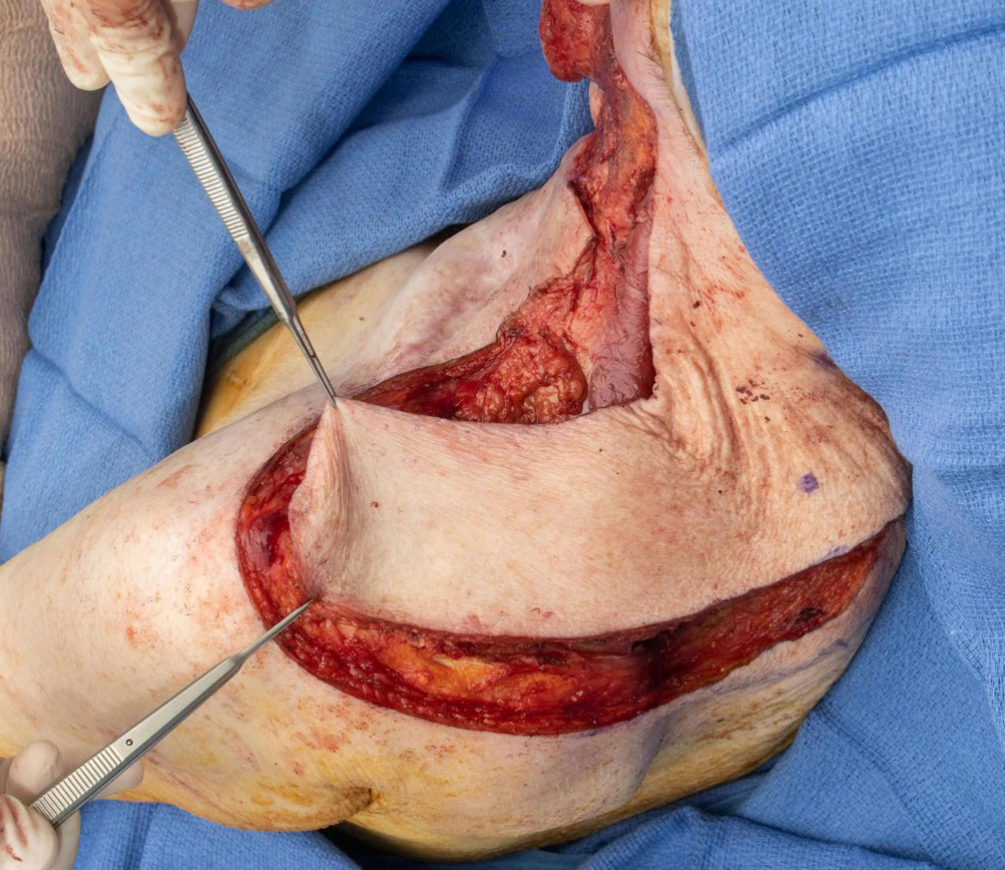
Superior trapezius flap (harvested)

**FIGURE 19:**
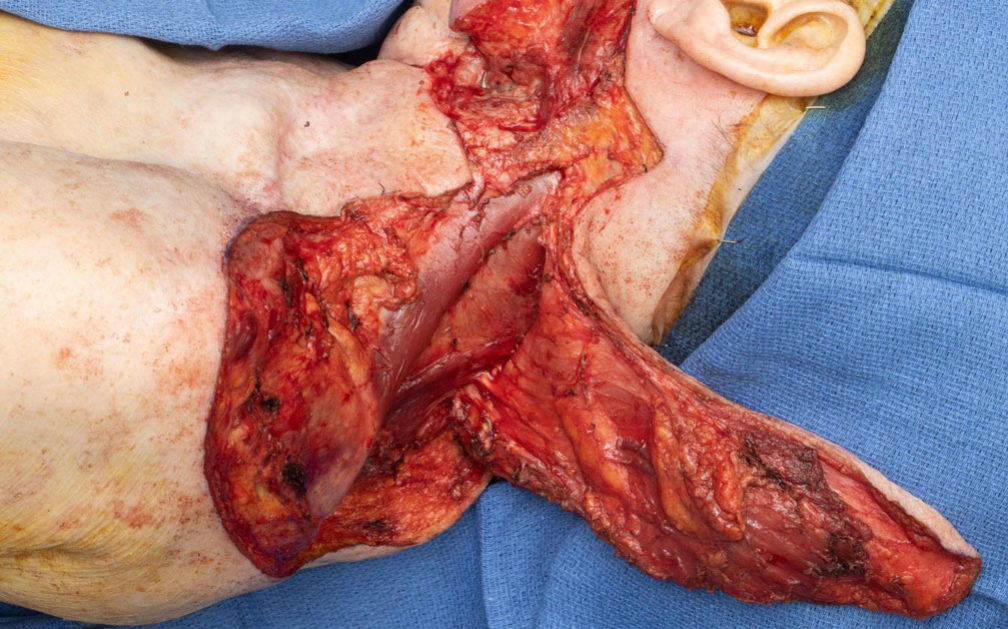
Superior trapezius flap (harvested and reflected)

**FIGURE 20:**
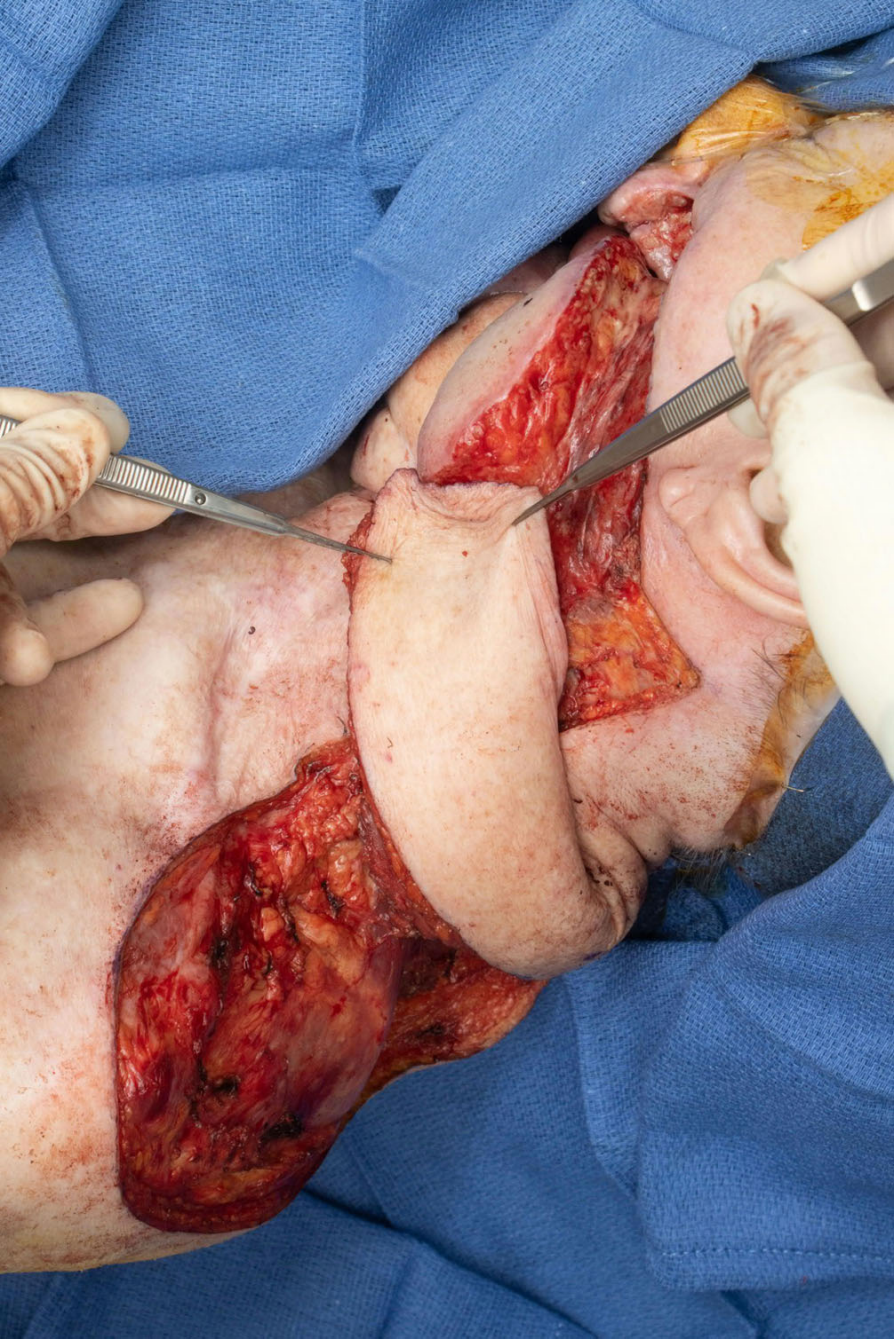
Superior trapezius flap (harvested and transposed toward defect)

**FIGURE 21:**
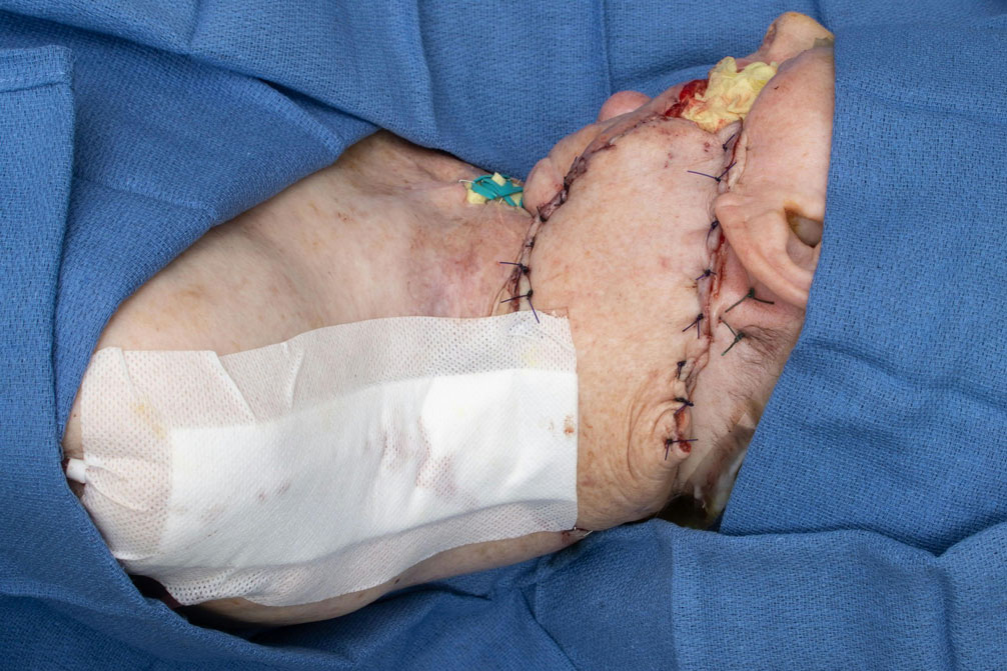
Superior trapezius flap (inset)

While all three of the trapezius system flaps have a potential role in salvage defects of the head and neck, some have more applicability than others. The lateral island trapezius flap is no longer commonly used, due to the advent of similar more accessible and reliable options (i.e., the supraclavicular and submental flaps). Prior treatment is a major consideration; caution is strongly advised if considering a lower island or lateral island trapezius flap due to their dependence on the transverse cervical vessels, which may have been sacrificed in previous neck dissections. The superior trapezius flap does not depend on transverse cervical vessels, and so its blood supply is likely to still be available in most head and neck cancer patients (Figures 18 and 19). This makes it especially useful for situations such as carotid exposure or recalcitrant wounds. However, the contour abnormalities associated with transposition of this flap can make it less cosmetically favorable (Figures 20 and 21). The arc of rotation of the superior trapezius flap is limited to coverage of lateral and posterior neck defects and it requires skin grafting to the donor site.

## 8 PEDICLED OSSEOUS RECONSTRUCTION

Large defects of the head and neck often involve bony structures, such as the mandible and maxilla. Osseous reconstruction typically heavily favors free tissue transfer. The fibula free flap, scapular free flap, and iliac crest free flap are most commonly used in the head and neck. Certain pedicled flaps can be harvested with limited osseous components and their utility will depend on the size and location of the bony defect.

The subscapular system is an extremely versatile donor site for head and neck reconstruction. Potential osseous options exist even for pedicled flaps, but arc of rotation limits their utility in the head and neck. Vascularized anterolateral rib, for example, can be harvested with serratus anterior based off the thoracorosal system but will not reliably reach the anterior mandible. The latissimus can also be harvested with 12th rib but transferring such a flap through the necessary tunnel is a challenge to the rib vascularity if the pectoralis major flap has not been previously harvested. The lateral trapezius flap can be harvested with scapular spine providing adequate bone stock for lateral mandible if the vascular pedicle has been preserved.^54^ As mentioned above, the submental flap can be harvested with the bone of the mentum for pedicled facial reconstruction,^55^ but this is rarely enough bone to salvage any previous osteocutaneous free flap.

While osseous pedicled reconstruction is remotely possible, the ability to properly contour and position any donor bone is severely limited. Reconstruction may also be hampered by a limited volume of bone or an inability to get the bone to reach the defect. Such flaps are not encouraged and should only be employed with very careful forethought and planning. The advantages and disadvantages of having bony reconstruction should be carefully weighed in a salvage setting. In many cases, salvage outcomes may be better with soft tissue only used for reconstruction.

## 9 CONCLUSION

In summary, the regional pedicled flaps reviewed here including the pectoralis major flap, deltopectoral flap, supraclavicular flap, submental flap, latissimus flap, and trapezius flap are all viable options for large salvage defects in head and neck reconstruction. Each has its advantages and disadvantages and therefore the choice of flap should always be tailored to the specific patient needs and the nature of the defect and not to the reconstructive surgeon's limitations (Table 1). We stress the importance of having proficiency with these flaps thereby ensuring the surgeon's possession of a full “reconstructive toolbox,” particularly for large‐sized salvage setting defects.

**TABLE 1:** Comparison of pedicled flap characteristics

Flap	Ease of harvest	Bulk	Surface area	Donor site morbidity	Reach
Pectoralis major	*****	*****	****	***	**
Deltopectoral	*****	**	****	**	*
Supraclavicular	****	*	**	*	***
Submental	***	***	***	*	****
Latissimus dorsi	***	*****	*****	***	*****
Trapezius	***	****	***	****	***

*Note*: *, least favorable; *****, most favorable.

## FUNDING INFORMATION

None.

## CONFLICT OF INTEREST

None.
